# Editorial: Adaptive Gait and Postural Control: from Physiological to Pathological Mechanisms, Towards Prevention and Rehabilitation

**DOI:** 10.3389/fnagi.2020.00045

**Published:** 2020-02-25

**Authors:** Helena M. Blumen, Paolo Cavallari, France Mourey, Eric Yiou

**Affiliations:** ^1^Departments of Medicine and Neurology, Albert Einstein College of Medicine, The Bronx, NY, United States; ^2^Human Physiology Section of the DePT, Università degli Studi, Milan, Italy; ^3^Laboratory “Cognition, Action et Plasticité Sensorimotrice,” INSERM U1093, Burgundy University, Dijon, France; ^4^CIAMS, Université Paris-Saclay, Orsay, France; ^5^CIAMS, Université d'Orléans, Orléans, France

**Keywords:** posture, gait, aging, prevention, rehabilitation

## Introduction

Gait and postural control are affected by aging, and in neurological, and musculoskeletal disorders. Poor gait and postural control are associated with disability, falls, increased morbidity and mortality; therefore representing major public health issues. The aims of this Research Topic was two-fold. First, it aimed to promote a better understanding of the patho-psychophysiological mechanisms affecting posture and gait in normal and pathological aging. Second, it aimed to provide an up-to date picture of motor and cognitive interventions directed to restore posture and gait in different aging populations. This Research Topic includes 29 contributions (16 original articles, 2 reviews, 3 systematic reviews, 5 clinical trials, 1 perspective, 1 methods paper, and 1 brief report) which, as a whole, report investigations related to posture and gait in several different populations, including healthy young and older adults, individuals that fall or have preclinical stages of dementia, and patients with stroke or Parkinson's disease—through the use of multidisciplinary concepts and techniques including Biomechanics (Neuro)physiology, Neuroimaging and Psychology. These contributions were subdivided into three key sections: (1) posture and gait changes during normal and pathological aging; (2) motor and cognitive preventive/rehabilitative interventions to restore posture and gait; (3) evaluation of posture and gait in normal and pathological aging.

## Posture and Gait Changes During Normal and Pathological Aging

### Changes in Sensory-Motor Integration and Postural Control During Normal and Pathological Aging

The ability to combine information across sensory modalities is an integral aspect of mobility, and has been examined by analyzing the role/use of proprioceptive and visual information. Whole-body vibration (WBV) is a training method used by clinicians to improve specific motor outcomes in various populations, such as old and young adults. In this regard, Delafontaine et al. showed that WBV applied prior to gait initiation increases stance leg stiffness, an effect known to be detrimental to stability. This negative effect, however, was compensated for by an increase in the amplitude of “anticipatory postural adjustments,” resulting in improved stability. These changes were observed in young adults, but not old adults. These findings are consistent with the hypothesis that balance control mechanisms within the postural system are interdependent (i.e., they may compensate each other if one component is perturbed) and vary with age.

Asymmetrical sensory-motor function after stroke creates unique challenges for bipedal tasks such as walking or perturbation-induced reactive stepping. Martinez et al. examined perturbation-induced stepping between legs, in stroke survivors and non-impaired controls. Participants were given an anterior perturbation from three stance positions: symmetrical distribution of the body weight or asymmetrical distribution on the preferred and the non-preferred leg. They found that stepping with the more-involved leg can be facilitated by un-weighting the leg. Post-stroke participants had earlier anticipatory postural adjustments, and always took more steps than controls to regain balance. These differences point to the simultaneous bipedal role (support and stepping) both legs have in reactive stepping.

When it comes to visual information, people can quickly adjust goal-directed hand movements to an unexpected perturbation, such as a target jump, or background motion. Zhang et al. examined whether this ability correlates with age. Although young and old adults were equally accurate, reaction time, and movement times were longer in old adults, and the responses were less vigorous. Moreover, old adults responded more strongly to the motion of the background than to the target jump. Thus, old adults showed delayed responses to visual perturbations and relied more on visual surroundings to adjust goal-directed movements. Given the observed overlap in neural circuitry necessary for both multisensory integration and goal-directed locomotion, it has also been shown that visual-somatosensory integration is significantly associated with gait pace but not rhythm, which is a more automatic process controlled mainly through brainstem and spinal networks. Actually, Mahoney and Verghese demonstrated that in old people stride length variability, but not swing time, is associated with magnitude of visual-somatosensory integration, concluding that worse visual-somatosensory integration in aging is associated with worse spatial, but not temporal, components of gait.

Lower-limb intersegmental coordination is a complex component of human walking, and aging may result in impairment of motor control and coordination contributing to the decline in mobility, and inducing loss of autonomy. In this regard, two reviews, by Tesio and Rota and by Delafontaine et al. were devoted to the analysis of center of mass (CoM) during walking and to anticipatory postural adjustments during gait initiation in stroke patients. Tesio and Rota state that the trajectory of CoM is a promising summary index of both balance and the neural maturation of walking. In fact, alterations in CoM motion could reveal motor impairments that are not detectable by clinical observation. In asymmetric gaits, the affected lower limb avoids muscle work by oscillating almost passively, but extra work is required from the unaffected side and the average work across a stride remains normal. In more demanding conditions, the affected limb can provide more work; however, the unaffected limb does also, and asymmetry between the steps persists. This “learned” asymmetry is a formerly unsuspected challenge to rehabilitation attempts to restore symmetry. The study of the three-dimensional trajectory of the CoM motion, which assumes a figure-of-eight shape, also represents a clinical frontier. Shape and size of the “figure-of-eight" seems not to change from child to adulthood. Delafontaine et al. make clear that stroke patients undergo a decrease in the *tibialis anterior* activity associated with difficulties in silencing *soleus* muscle of the paretic leg, display a decreased shift of center of pressure, and have lower propulsive anterior forces and suffer of a longer preparatory phase. Regarding possible gait-rehabilitation strategies, this systematic review suggests that the use of the non-paretic as the leading leg can be a useful exercise to stimulate the paretic postural muscles. Moreover, Gueugnon et al. evaluated the impact of aging on the coordination of lower limb kinematics and kinetics during walking at a comfortable speed. They showed that lower-limb coordination modifies with age, thus influencing ankle motion and power. They also hypothesized that this modification of coordination constitutes a neuromuscular adaptation of gait control in order to maintain gait efficiency. Foot-shank coordination might thus represent a valid outcome measure to estimate the efficacy of rehabilitative strategies and to evaluate their efficiency in restoring lower-limb synergies during walking.

Tripping over an obstacle may end with a fall, but a fall can also be caused by a spontaneous loss of balance, which are common during normal, and pathological aging. Thus, fall prevention is an important topic. A study by Jayakody et al. examined changes in gait variability—a known predictor of falls—factors (medical, sensory-motor, cognitive, and demographic) that may predict this variability across time (close to 5 years). They found that variability in different gait measures do not undergo uniform changes. Furthermore, each variability measure was associated with different factors such as presence of cardiovascular disease, arthritis and body mass index, quadriceps strength, postural sway, processing speed, and lower education. These results provide useful information on potential targets for future trials to maintain mobility and independence in aging. The risk of falling, and the changes in balance control strategy, was further analyzed by Li et al. More specifically, they compared the differences in the kinematic characteristics of crossing obstacles of different heights between stroke survivors and age-matched healthy controls. The authors suggested that, because of reduced muscle strength, stroke survivors use a conservative strategy to negotiate the obstacles and control balance. As a result, abnormal patterns during obstacle crossing increase the risk of falling. They propose that future rehabilitation training program should be designed to enhance body stability, reduce energy cost, and improve motion efficiency.

A better understanding of the pathophysiology of steering, which requires an interaction between posture, balance and progression along non-linear trajectories, is reviewed by Godi et al. Their review focused on the fundamental processes (physical laws, changes in muscle/brain activation, role of proprioception) allowing for changes in locomotion trajectory along a curved path in old adults, and people with Parkinson's Disease (PD). They also propose rehabilitative approaches to improve locomotion in order to reduce the risk of falling in both healthy old adults and patients. Finally, Lu et al. evaluated the timing of avoidance of a virtual obstacle in old patients with PD in the off-medication state and in young and old controls, while walking on a treadmill. They found that the probability of successful obstacle avoidance was associated with the timing of obstacle appearance, and that age was positively correlated with the time required to successfully avoid obstacles. Nonetheless, the PD group required significantly more time. They concluded that slowing of gait adaptability could contribute to high fall risk in old adults and PD because of disturbances in motor planning, movement execution, or disordered response inhibition.

### Relationship Between Psycho-Cognitive Conditions and Motor Control of Gait in Normal and Pathological Aging

Walking is a highly automated process, but as we age the demands for controlled processes increases. Walking is also influenced by psychological factors such as fear of falling (FOF). Several articles in this topic focuses on psycho-cognitive conditions and control of gait during normal and pathological aging.

Dual-tasking (DT; walking while performing a secondary task) paradigms are often used to examine the cognitive demands of walking, as it involves attention switching and task prioritization. However, studies using this paradigm to explain age-related performance decline sometimes reveal contradictory results. To clarify this point, Wollesen and Voelcker-Rehage investigated whether differences in demographics, physical functioning, FOF, and other psychological factors could explain reduced DT performance. Their results confirm a large inter-individual variability in old adults, suggesting that factors causing performance differences in DT costs need to be reassessed. Specifically, functional age may be a better predictor of DT costs than chronological age, and psychological factors have a negative effect on DT performance.

The neural correlate of DT costs in old adults was investigated by Poole et al. who showed that faster walking was associated with increased functional connectivity between motor and cognitive networks, and decreased functional connectivity between limbic and cognitive networks. Moreover, smaller DT costs was associated with increased connectivity within the motor network, and increased connectivity between ventral attention and executive networks. These findings support the importance of both motor network integrity as well as inter-network connectivity amongst higher-level cognitive networks in healthy old adults' ability to maintain mobility, particularly under DT conditions. Hermand et al. focused on examining prefrontal cortex (PFC) activity of subacute stroke patients in DT situations. Their results showed a “ceiling” effect in PFC oxyhemoglobin levels while walking, leaving no available resources for simultaneous cognitive tasks during the early recovery period following stroke. In these patients, cognitive, but not motor performance declined with a higher cognitive load, i.e., prioritization was given to maintain walking performance. Interestingly, a similar finding was reported in healthy old adults by Vervoort et al. who examined the effects of aging and task prioritization on split-belt gait adaptation with or without a cognitive load. They showed that young and old adults modify, respectively, the perturbed leg's timing and gait speed to adapt to split-belt gait perturbations. Results also showed a decline in cognitive task performance during early gait adaptation, which suggests task prioritization, especially in older adults. They concluded that healthy old adults retain the ability to adapt gait to split-belt perturbations, but through a different adaptation strategy.

The ability to adapt gait to environmental constraints may also change in PD, as shown in the study of Caetano et al. These authors investigated cognitive, physical, and psychological factors associated with gait adaptation to obstacles and stepping target negotiation. They showed that executive function and reactive balance capacity are important for precise foot placements; and that cognitive capacity is important for step length adjustments associated with obstacle avoidance. The role of psychological factors on gait is also stressed by Santos Bueno et al., who investigate gait patterns in healthy old women with and without fall-history, and with high and low FOF. They found that after exposure to a fictional disturbing factor (psychological and non-motor agent), all participants had shorter step length, stride length, and slower walking speed. Those without fall-history and with high FOF, however, showed greater changes and lower Gait Profile Score. They concluded that these gait changes in the presence of a FOF causing agent led to a cautious gait pattern in an attempt to increase protection.

## Motor and Cognitive Preventive/Rehabilitative Interventions to Restore Posture and Gait

A better understanding of the pathophysiological mechanisms associated with gait and posture can ultimately be used to develop successful interventions for maintaining gait and posture in healthy and pathological aging. This Research Topic incorporates articles describing interventions that aim to maintain gait performance in healthy old adults, in individuals with chronic and subacute stroke, and in old adults with mild cognitive impairment (MCI).

In a study of healthy old adults, Eckardt and Rosenblatt investigated how different resistance training modalities affect kinematic synergies related to the medio-lateral trajectory of the swing-foot during normal and perturbed gait. Researchers found that training using unstable conditions had a significant effect on kinematic synergy. They concluded that unstable training conditions promote neuromuscular adaptations and should be incorporated into training programs.

The relative efficacy of 4 weeks of underwater gait training with water jet resistance and underwater gait training with ankle weights was explored in 22 middle-aged to old adults with chronic stroke by Lim. Results indicated that underwater gait training with water jet resistance led to greater improvements in dynamic balance than underwater gait training with ankle weight, as well as a number quantitative gait measures (e.g., gait velocity, cadence, and step length). In another pilot RCT of 37 individuals with subacute stroke, Lim  examined the relative efficacy of multi-sensory training and treadmill gait training on proprioception and balance. After 8 weeks of training, conventional rehabilitation, and multi-sensorimotor training generated more improvements than conventional rehabilitation and treadmill gait training on both proprioception and balance—encouraging results for designing a future large-scale RCT. Another contribution to this topic (Giannouli et al.) presents a conceptual framework that is ripe for exploring the efficacy of a novel square-stepping program for falls prevention in older adults.

The benefits of a suite of computer games that involves physical exercise and demands different cognitive functions was explored in old adults by de Bruin et al. Quantifying EMG coherence and gait during single and dual-task walking conditions, they found that 6 weeks of exercise improved corticospinal transmission to the *tibialis anterior* muscle, and increased minimum toe clearance during dual-task walking conditions. These preliminary findings are encouraging, but need to be more systematically examined in a randomized controlled trial (RCT). The relative efficacy of virtual-reality based and traditional physical and cognitive training was explored in an RCT of 34 older adults with MCI by Liao et al. Results suggest that virtual-reality based cognitive and physical training leads to greater improvements in dual-task gait performance (cadence) and a measure of executive function (trail making test) in MCI. Others (Pedroli et al.) propose a virtual-reality based motor rehabilitation program for falls prevention in frailty that takes place in a CAVE system (4-screen room with a stationary bike) and focuses on improving balance.

A final contribution to this section is a systematic review by Fischer et al. of WBV training for balance control in healthy and pathological participants over the lifespan (16 studies evaluated the effects in old adults). They concluded that there was a positive effect of WBV on gait speed and the timed-up-and-go test in old adults, but a consensus on specific WBV training was not reached.

## Evaluation of Posture and Gait in Normal and Pathological Aging

A better understanding of the pathophysiological mechanisms associated with gait and posture is also dependent on appropriate measures and characterization of at-risk individuals. In this Research Topic, several articles address these two related issues. Mandigout et al. compared step counts of wrist-worn and hip-work actigraphs in 22 young and 22 old adults over a 24-h period. Their key finding was that step counts are greater from wrist-worn actigraphs than hip-worn actigraphs in both young and old adults. In a systematic review of young adults, old adults and PD patients, Siragy and Nantel found that quantifying dynamic balance can be challenging due to varying measures and definitions, reflects neuromuscular stability mechanisms, and depends on whether walking conditions are perturbed or unperturbed. Thus, caution is recommended when comparing step counts from different actigraphs and different measures of dynamic balance across different studies.

Caution is also recommended when characterizing old adults with the motoric cognitive risk (MCR) syndrome. The motoric cognitive risk syndrome is a preclinical stage of dementia that is typically characterized as slow gait speed (for their age and sex) and a subjective cognitive complaint (Verghese et al., 2013). In this Research Topic, Sekhon et al. examined if increased five-times-sit-to-stand time rather than slow gait speed could be used to characterize individual with MCR. They found that the prevalence of this new characterization of MCR is lower than the typical characterization of MCR and that it does not predict non-amnestic MCI—thus, may not be tapping in to the same preclinical stage of dementia.

Finally, Corzani et al. characterized motor adaptation in response to acoustic stimuli during walking in individuals with PD. In order to improve gait rhythmicity their interdisciplinary team developed wearable technologies to stimulate gait rhythm and increase or decrease cadence. Their results suggest that verbal cuing is more effective at restoring normal cadence than metronome beats.

## Author Contributions

HB, PC, FM, and EY drafted, revised, and approved the final version of manuscript.

### Conflict of Interest

The authors declare that the research was conducted in the absence of any commercial or financial relationships that could be construed as a potential conflict of interest.
